# Optimization Complete Area Coverage by Reconfigurable hTrihex Tiling Robot

**DOI:** 10.3390/s20113170

**Published:** 2020-06-03

**Authors:** Anh Vu Le, Rizuwana Parween, Rajesh Elara Mohan, Nguyen Huu Khanh Nhan, Raihan Enjikalayil Abdulkader

**Affiliations:** 1 ROAR Lab, Engineering Product Development, Singapore University of Technology and Design, Singapore 487372, Singapore; leanhvu@tdtu.edu.vn (A.V.L.); rizuwana_parween@sutd.edu.sg (R.P.); rajeshelara@sutd.edu.sg (R.E.M.); raihan619@gmail.com (R.E.A.); 2 Optoelectronics Research Group, Faculty of Electrical and Electronics Engineering, Ton Duc Thang University, Ho Chi Minh City 700000, Vietnam

**Keywords:** shapeshifting robot, tiling robotic, path planning, complete coverage, energy optimization

## Abstract

Completed area coverage planning (CACP) plays an essential role in various fields of robotics, such as area exploration, search, rescue, security, cleaning, and maintenance. Tiling robots with the ability to change their shape is a feasible solution to enhance the ability to cover predefined map areas with flexible sizes and to access the narrow space constraints. By dividing the map into sub-areas with the same size as the changeable robot shapes, the robot can plan the optimal movement to predetermined locations, transform its morphologies to cover the specific area, and ensure that the map is completely covered. The optimal navigation planning problem, including the least changing shape, shortest travel distance, and the lowest travel time while ensuring complete coverage of the map area, are solved in this paper. To this end, we propose the CACP framework for a tiling robot called hTrihex with three honeycomb shape modules. The robot can shift its shape into three different morphologies ensuring coverage of the map with a predetermined size. However, the ability to change shape also raises the complexity issues of the moving mechanisms. Therefore, the process of optimizing trajectories of the complete coverage is modeled according to the Traveling Salesman Problem (TSP) problem and solved by evolutionary approaches Genetic Algorithm (GA) and Ant Colony Optimization (ACO). Hence, the costweight to clear a pair of waypoints in the TSP is defined as the required energy shift the robot between the two locations. This energy corresponds to the three operating processes of the hTrihex robot: transformation, translation, and orientation correction. The CACP framework is verified both in the simulation environment and in the real environment. From the experimental results, proposed CACP capable of generating the Pareto-optimal outcome that navigates the robot from the goal to destination in various workspaces, and the algorithm could be adopted to other tiling robot platforms with multiple configurations.

## 1. Introduction

Tiling robots executing the regular and tedious tasks in the cleaning and maintenance industry have arisen significantly. These robots play the role of useful tools to ease the manual workload. A recent survey shows that the market for service robots is growing rapidly and is forecast to reach 4.34 billion USD by 2023 [1]. The world’s leading technology companies, especially those providing e-commerce services such as Amazon, Alibaba, DHL, Tesla, and Google, have invested considerable resources in developing their robots. These robotic models have operated gradually in large and complex environments and proved that they could archive automated operations that help to reduce production and operating costs [2].

A tiling robot consists of various mechanical components such as motors, links and joints, as well as electronic components such as sensors and drivers, that need to be well corroborated to fulfill the autonomous navigation [3]. For instance, to enable the perception environment during navigation, recent robots are equipped with perception sensors units such as LiDAR, 3D camera, wheel encoders, and Inertial Measurement Unit (IMU)to power the Simultaneous localization and mapping (SLAM) [4] capabilities. To ensure the self-localization in the global cluster and dynamic environment with various sensor inputs, the use of efficient sensor fusions such as Kalman filter and particle filter to reduce the sensor noise has been studied intensively. Besides, bump sensors and time of flight sensors help the robot to identify the static and dynamic obstacles in the local frame and plan the optimal path to navigate smoothly from source to goal. The hardware setup should provide the options to ease the robot to understand the working conditions and to respond effectively in the real clutter working environment [5]. Together with the novelty sensor fusion algorithms, the appropriate combination of local path planner and globe path planner determines the performance of the robot navigation [6,7].

The CACP focuses on generating the global path planner to maximize the space visited by tiling robots inside the defined working environments. This task involves trajectory generating and obstacle avoidance to cover the whole area in the considerations of a safe journey, effective energy consumption, and time saving. The CACP algorithms have been developed for static and dynamic working conditions. Cell-based decomposition [8], Morse-based cell partition [9], hypothesis based on diagrams [10], and 3D mesh decomposition [11,12] are used to simplify the workspace before applying CACP algorithms. For CACP in the grid-based workspace, several authors have proposed various optimal global plans to work with tiling robot, for example frame-based separation [13], wavefront [14], neural network [15], and a tetrominal method [16]. The hypothetical cell decline suggested by Choset [17] is the best-known approach among the conventional strategies because of its adaptability in adjusting to specific situations. This approach separates the grid-based map to smaller sub-maps. After defining the sub-map with the complexity indication in each sub-workspace, the approach creates a route path that covers the whole predefined area with the help of simple path planning algorithms such as zigzag. This idea has been applied for cleaning robots [18].

It is worth noting that the conventional CACP approaches have been verified extensively on robots with fixed shape. Because of the fixed shape constraint, these robots do not have other options to automatically explore working areas and define the optimal path to avoid obstacles during the area coverage. Reconfigurable tiling robots that can shift their shapes flexibly are an effective solution to overcome these challenges. The motivation from the fact that the bigger is the size of the robot base frame, the faster it will cover the workspaces. However, a large base size makes it difficult for a robot to reach narrow spaces. Therefore, the robot can access restricted space areas with the ability to change its size into different base frame levels. Besides, changing shape helps to link the predefined tilesets from source to destination with the shortest optimal path, which ensures the complete coverage, saves the navigation energy and time. To this end, a shape-shifting strategy was proposed in our previous works [19,20] for the novel robot named hTetro. Those works validated the application of polyomino tiling theory in the context of area coverage and showed the superior area coverage with the involvement of the shape-shifting robot in the narrow constraint conditions. However, the generation of the tileset and the robot’s coverage process was executed by manual supports without any motion planning strategies. Another previous work proposed a reconfigurable Polyiamond shape-based robot with four blocks have given seven shape-shifting mechanisms [21]. In this work, the design of the platform was described in detail. However, the locomotion is entirely different, which is omnidirectional-based locomotion.

There are several methods used to find the shortest path connecting source to destination using reconfigurable robots. For instance, revised GA with customized fitness functions are implemented to solve the path planning problem of the lattice modules in M-Lattice robot [22]. To plan the path to overcome stairs or obstacles, KAIRO 3 robot makes use of extended RRT* algorithm [23] to autonomously calculate the actions required for the tasks [24]. Research has also been conducted to provide heuristic-based algorithms [25] and distributed planning algorithms [26] for lattice-type inter-reconfigurable robots that are less architecture-specific. Since the ability to shape-shifting and interact within obstacles of the reconfigurable class of tiling robot is unique to fix form robots, applying the conventional CACP is not straightforward. Due to the intrinsic complexity of reconfigurable robots, autonomous path planning between different configurations has been a complicated topic, and furthermore CACP with multiple configurations involved is even more challenging. With the increased degree of freedoms in reconfigurable robots and the additional constraints due to different robot configurations, simple CACP approaches are no longer sufficient to determine optimal solutions; therefore, new or revised path planning approaches have been designed to tackle CACP problems for each reconfigurable robots based on the possible topology and the available motions.

TSP is the well-known approach to find the path linking all the tiles within the generated tileset. Solving the path by TSP optimization requires a specific fitness function to be derived according to the robot platform’s behavior to connect a pair of waypoints. Finding the optimal solution of TSP requires a vast number of calculations. Finding the solution for TSP is an non-deterministic polynomial-time NP-hard problem where the complexity will increase exponentially when increasing the nodes in TSP [27]. It is a costly effort by brutal search to create the ideal solution with TSP. In particular, there are (n(n−1)!)/2 possible trajectory solutions for *n* nodes. Simple operations, for example, spiral and zigzag patterns, have been taken in a conventional cleaning robot with TSP [28], but these are not energy efficient. Evolutionary algorithm-based complete coverage path planning is the feasible solution to compromise the time expense and the short path searching. GA [29] and ACO [30] are the most well-known evolutionary methods to solve the TSP. These methods are based on an integrated learning process through the change from a group of individuals [31]. By constantly running change processes, compositing, and selection, the algorithm can create ideal arrangements quickly, even in a larger workspace.

This work presents the novel CACP framework for a shape-shifting tiling robot with three hexagon shape-based modules called hTrihex. The proposed robot is considered as inter reconfigurable platform where changing the morphologies is done by the active servo motors located at robot hinges. The developed CACP-based tiling theory guarantees to generate the optimal tileset for confined workspaces. We are then finding the sub-optimal path for predefined hexagon-based tileset by GA and ACO methods. In addition, because of the reconfigurable abilities of the introduced platform, the energy costweight during routing the pair of honeycomb base tilesets is considered as the unique actions during reconfiguration and locomotion, including linear moving, heading correction, and transformation.

The main contributions of this paper are threefold. First, we propose an evolutionary optimization-based CACP approach for hTihex reconfigurable tiling robots, which is represented in hexagon-based grid workspace. Second, we develop the TSP costweight, which is dependent on the vitality profile of each activity of the hTrihex robot during operation. Third, the proposed CACP was tested on the real robot platform, proving the efficiencies in terms of energy consumption and travel time.

The paper is organized as follows. The architecture of hTrihex is introduced in Section 2. The description of the proposed robot on the lattice workspace is detailed in Section 3. In Section 4, the proposed CACP method for the hTrihex platform by the tiling hypothesis is presented. The optimal CACP of the proposed strategy is validated in Section 5. The conclusion, together with potential future works, is explored in Section 6.

## 2. The hTrihex Robot Description

**Figure 1:**
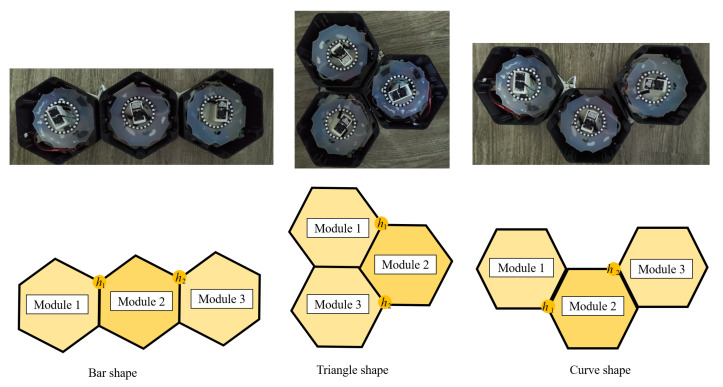
hTrihex reconfigurable robot with three hexagon-based morphologies.

The structure of the beehive inspires the design principle of the hTrihex robot. The robot consists of three hexagonal modules connected by two active hinge links. The hinges can freely rotate 0−4π/3 in the area from the edge of the honeycomb module to create three morphologies named Triangle, Bar, and Curve, as can be observed in Figure 1. From one of the three robot configurations, it is possible to switch to the desired configuration by changing the rotation angle and direction of servo motors at the joints between robot modules.

**Figure 2:**
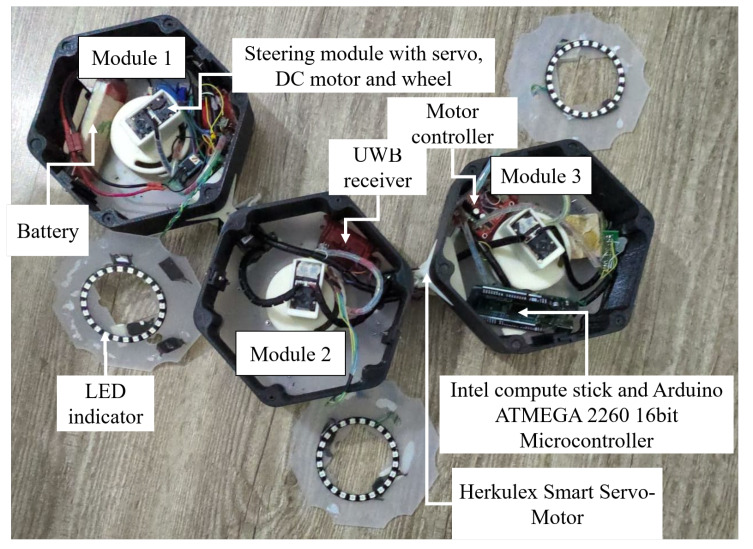
Electronic components of hTrihex.

**Figure 3:**
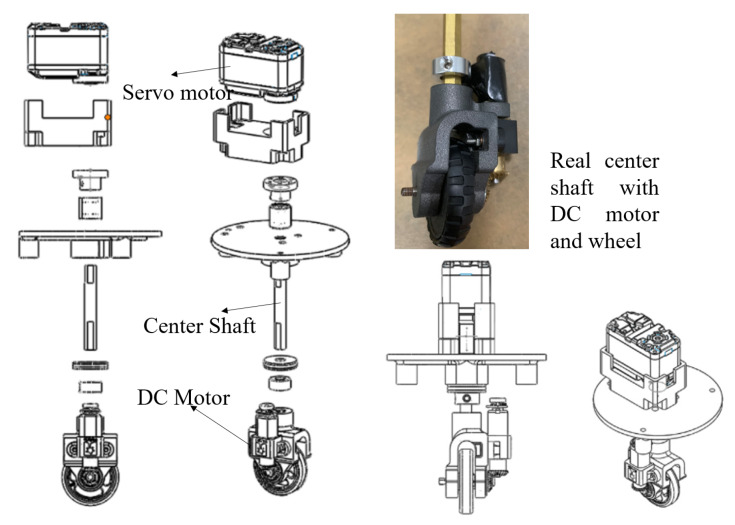
Independently-controlled steering and driving Locomotion module.

Figure 2 describes the electronic components located in each robot module. Since the robots can change their shapes among three available morphologies, synchronizing the moving of modules corresponding to each robot shape is required for the smooth locomotion. To this end, an independently-controlled steering and driving locomotion unit, as shown in Figure 3, consisting of a navigational servo motor and a direct current (DC) motor responsible for locomotion is mounted on each module. To carry out the movement, each locomotion module is equipped with a servo motor that can change to any desired angle within 0–2π rad around the center shaft. The robot can monitor the servo motor’s current steering angle during navigation. After deciding which direction to move in any given shape, the three servo motors automatically synchronize together in that direction then activate the DC motors to move the robot. The 50-mm diameter wheel is mounted on each DC motor. The DC motor is selected with a plastic-geared (gear ratio of 200:1), 7.4 V, maximum torque of 1.37 Nm, and a maximum speed of 60 rpm to carry the robot mass. The servo motors at the robot hinge and locomotion units are Herkulex servos with the specs 7.4 V and torque of 3.52 Nm. The moving command is controlled through the central processing unit Intel Compustick to Roboclaw motion driver and an Arduino microcontroller.

The indoor positioning system by Ultra Wide Band UWB technology from the Marvelmind is used to provide an instant global position to the robot during the navigation. The UWB system can archive 2D location in the mobile system moving with the velocity of less than 5 m/s with accuracy in the range of ±2 cm. The information from the rotary wheel encoder will be integrated by the navigation time to provide the location in the local robot frame after the start of the navigation. The Extended Kalman Filter (EKF) solution is used to fuse all 2D x, y positions from Ultra Wideband UWB Beacons, wheel encoders, in combination with the IMU orientation sensor to provide odometry information. All hardware modules are controlled via Robot Operation System (ROS) [32]. An Intel compute Stick is used to plan the navigation path and transmit commands, including the desired shape, travel distance, and direction to the actuators. A battery of 14.4 V is the primary power source, and the appropriate regulators are used to distribute the power for electronic and manipulation components of the robot. Robot weight with all components is about 1.2 kg. The unique design allows the robot to navigate inside the hexagon grid-based workspace. The advances in the drive mechanism of the current reconfigurable robot platform compared with conventional omnidirectional designs are not the main topics to address in this paper. After introducing the kinematic design of a hTrihex with independently-controlled steering and driving locomotion module, we focus on representing the hTrihex platform inside the hexagon-based grid workspace and then proposing a navigation strategy that accomplishes complete path planning in the next sections.

## 3. Description of hTrihex Inside the Hexagon-based Grid Workspace

**Figure 4:**
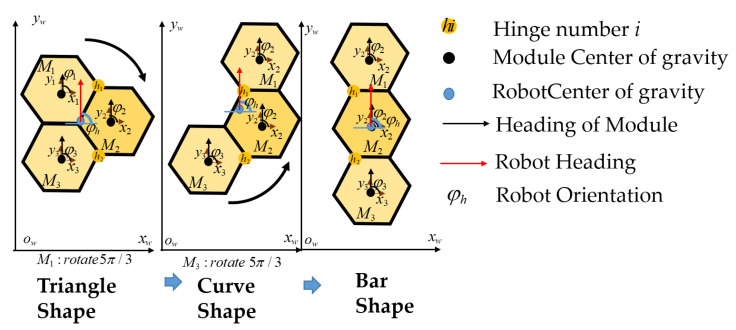
Description of hTrihex inside the workspace with shape shifting, heading, and orientation.

The predefined workspace *w* is partitioned into the regular hexagonal cells equal to the robot block. The center of the robot’s mass (COM) corresponding to each robot morphology is selected to represent the robot location as the predefined waypoint *W* in the workspace *w*. The module locations and the COM for each morphology among the Triangle, Bar, and Curve sets are depicted in Figure 4. In this figure, the operation to shift the shape robot from Triangle to Bar and then to Curve with the required angle of hinge ID among $h_{1},h_{2}$ rotation is also described. As the results, the waypoint at robot COM location as $x_{h}^{w},y_{h}^{w}$ and the robot orientation of module $M_{1}$ as $\varphi_{h}^{w}$ details the robot odometry in the working environment. The hTrihex module *i* is represented by $\left\{x_{i}^{w},y_{i}^{w},\varphi_{i}^{w}\right\}$, where *i* is the module ID of three robot module $\left(i\in \left\{M_{1},M_{2},M_{3}\right\}\right)$. The module mass is $m_{1},m_{2},m_{3}$, and the length from the point of rotation at the hinge to the COM of a specified module is $l_{m}$. The navigation trajectory of the robot in order to clear all the waypoints is divided into a number of pairs of two waypoints. For the trajectory with *N* waypoints, each pair in the trajectory is defined as $p(W_{k}^{s},W_{k}^{g})$, where *k* stands for the pair number, *s* is the source waypoint, and *g* is the goal waypoint. The initial waypoint has k=1, and the final waypoint has k=N−1. Note that, for the trajectory with *N* waypoints, there are N−1 pairs of waypoints, and for the workspace with N waypoints there is a set Ω which consists of N(N−1))/2 possible pairs of waypoints.

With the description of hTrihex inside the workspace, the proposed CACP framework will generate the path based on the predefined workspace and execute the robot to clear all the defined waypoints to ensure the complete coverage task.

## 4. CACP Framework for hTrihex Platform

### 4.1. Coverage Path Planning Based on hTrihex

The tiling theory was developed to create a solution that ensures a predefined workspace with the size that satisfies certain conditions and can be tiled entirely using the shapes of the hTrihex-based robot. The hTrihex platform tiles the pre-described environment with three configurations and the trihex-based tiling theorems are described as follows.The theorems can be proved in the same way as the theorems were proved for the rectangular-based platform hTetro [33].

**Figure 5:**
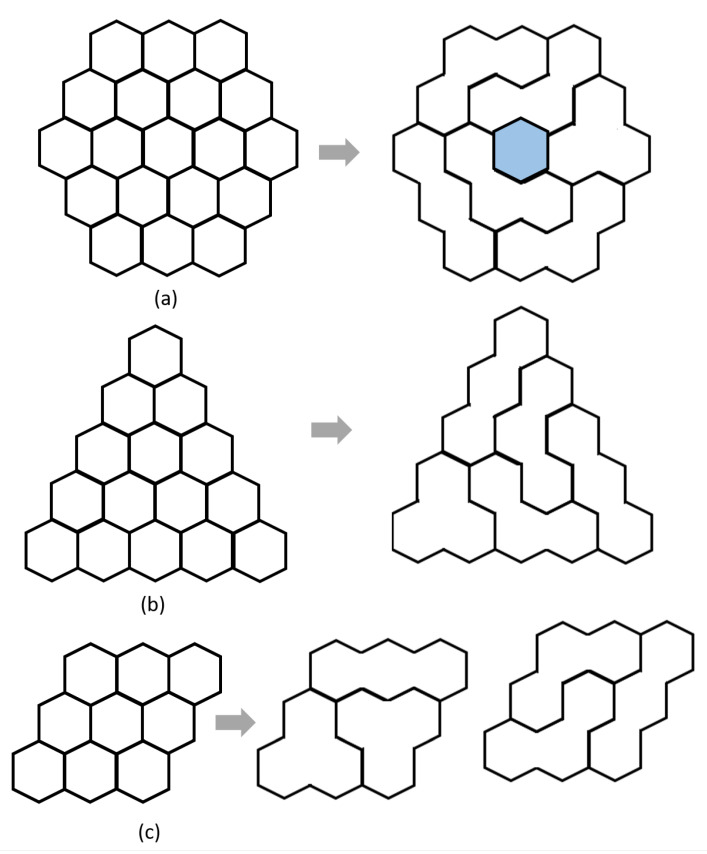
Different regular hexagon-based workspace in the form of: (**a**) ‘Honeycomb’; (**b**) ‘Hexagonal Triangle’; and (**c**) ‘Hexagonal Rectangle’.

**Theorem** **1.** *A honeycomb with side 3 consists of nineteen hexagons. It can be tiled with all the configurations of trihexes with a void at the center, as shown in Figure 5a.*

**Proof** **of** **Theorem 1.** One configuration of hTrihex consists of three hexagonal blocks, and the hTrihex is able to reconfigure three shapes. One honeycomb of side 3 consists of nineteen hexagons. Hence, a set of four curve shapes, one bar shape, and one triangle shape consist of eighteen hexagonal blocks, and this set covers a honeycomb with side 3 with a void space at the center. □

**Theorem** **2.** *A hexagonal triangle of side n consists of n(n+1)/2 triangles. It can be tiled with all the configurations of hTrihexes if (and only if) the total number of hexagons n(n+1)/2 is a multiple of three and n>3.*

**Proof** **of** **Theorem 2.** Hexagonal triangles of sides 4 and 5 consist of ten and fifteen hexagonal blocks, respectively. Among these, the hexagonal triangle with side 5 can be completely tilled by all forms of hTrihex, as shown in Figure 5b. The total number of hexagonal blocks in this hexagonal triangle is a multiple of three. In general, a hexagonal triangle of side *n* (n>3) consists of n(n+1)/2 hexagonal blocks. If the total hexagonal blocks n(n+1)/2 is a multiple of three, then the hexagonal triangle can be tiled with all the shapes of hTrihex. However, a hexagonal triangle of side n=3 consists of six hexagonal blocks, which is divisible by three. This triangle can be tiled with only two configurations (triangle and bar) of trihexes. □

**Theorem** **3.** *A hexagonal rectangle of side n consists of $n^{2}$ hexagons. It can be tiled with all configurations of hTrihexes if (and only if) the total number of triangles $n^{2}$ is a multiple of three and n>3.*

**Proof** **of** **Theorem 3.** A hexagonal rectangle of side 6 consists of thirty-six hexagons, and it can be filled with all forms of hTrihex. However, a hexagonal rectangle of side 3 consists of nine hexagons, as shown in Figure 5c, and it can be filled with only one form of hTrihex. □

**Figure 6:**
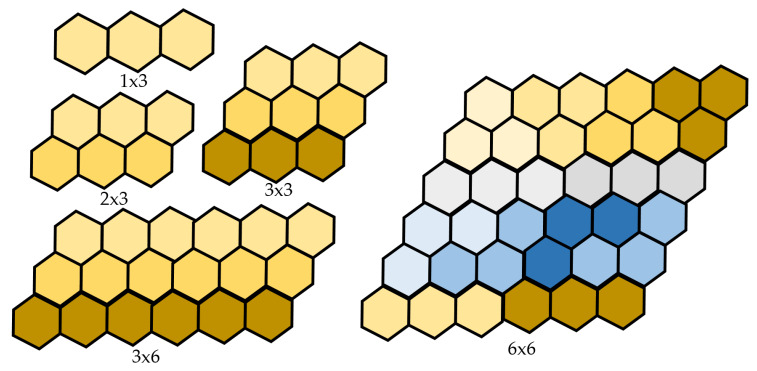
hTrihex covers the workspace by tileset.

**Theorem** **4.** *Any x × y can be covered with the combination of three morphologies, i.e., the Bar, Triangle, and Curve Trihexes, if (and only if) both x and y are multiples of 6, as shown in Figure 6.*

**Proof** **of** **Theorem 4.** Let ‘x’ and ‘y’ be the number of hexagon along both sides of the hexagonal grid workspace to be tiled. The area of the workspace of the hexagon of (x × y) parallelogram is xy/3. If (xy/2) = 3n (multiple of thee), where n is an integer number. Then, this workspace can be filled with Curve, Bar, and Triangle shapes entirely. The number of hexagons along each side of the hexagon must be larger than three. This guarantees that the proposed formulation of the theorem that any hexagon x × y can be filled with a set completely if and only if both x and y are multiples of 6. □

**Figure 7:**
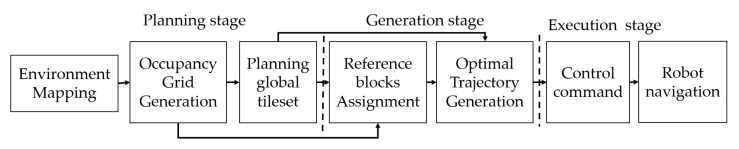
CACP framework for tiling robotics.

The structure of CACP in Figure 7 includes three states: planning, generation, and execution. After completing the tileset’s identification that needs to be placed to cover the map by tiling theory completely, using the backtracking algorithm [34], a random, unsigned tile is placed into the workspace. If the rollback loop is unable to arrange the next tiles, the other capabilities of the previous tile will be tried. The process is looped over until the predefined workspace is filled completely by robot shapes. The tileset plan with the robot shapes is depicted in Figure 6.

### 4.2. Assigning the hTrihex Module Location

**Figure 8:**
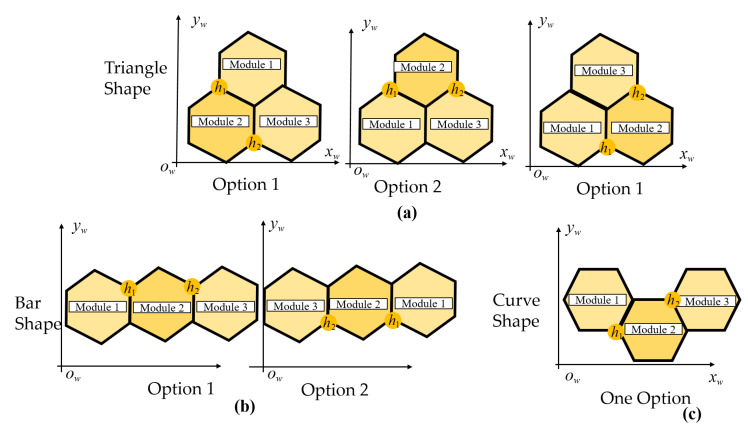
Module assignment for hTrihex inside the workspace: (**a**) Triangle shape; (**b**) Bar shape; and (**c**) Curve shape.

**Figure 9:**
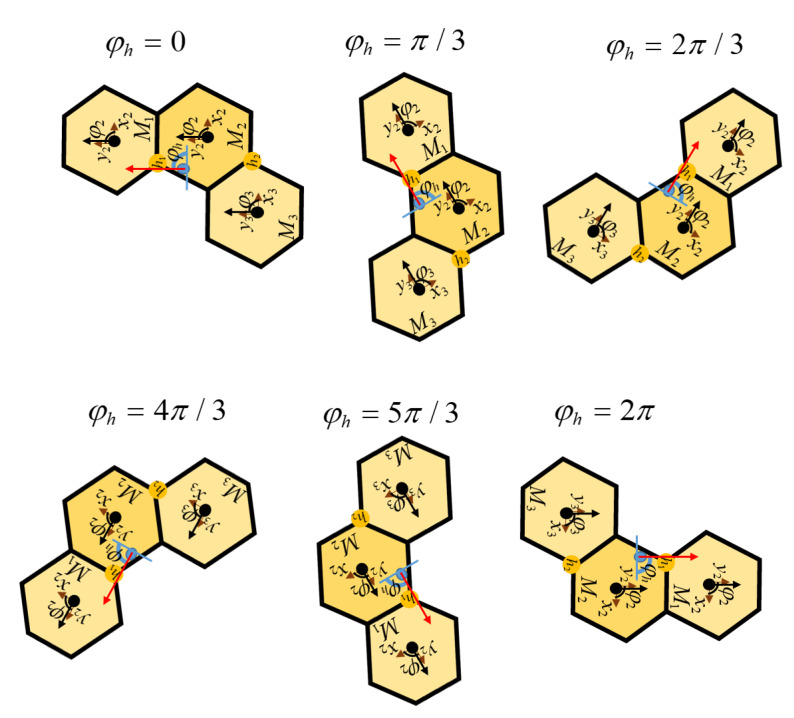
Module position inside the workspace corresponding with the heading of robot.

Tilesets based on the tiling theory only identify the overall robot three hexagons-based tiles inside the predefined grid-based environments. Considering each tile, the three modules with hexagonal shapes marked as $M_{1},M_{2},M_{3}$ can be located in different orders according to the arrangements of robot hinges; hence, these create the options for robot COM and change the route of CACP inside the workspace. Symmetrical Triangle and Bar shapes, as in Figure 8a,b, and the asymmetrical Curve shape, as in Figure 8c, yield different options for robot module locations, respectively. Because of the rotation directions of the servo motors at the robot hinges, there are two options to form the Triangle and Bar shapes with the same robot orientation. On the other hand, there is only one possibility to obtain the Curve shape in any specific robot orientation. Besides, the heading of robot will define the module location for each give tile, as shown in Figure 9.

**Figure 10:**
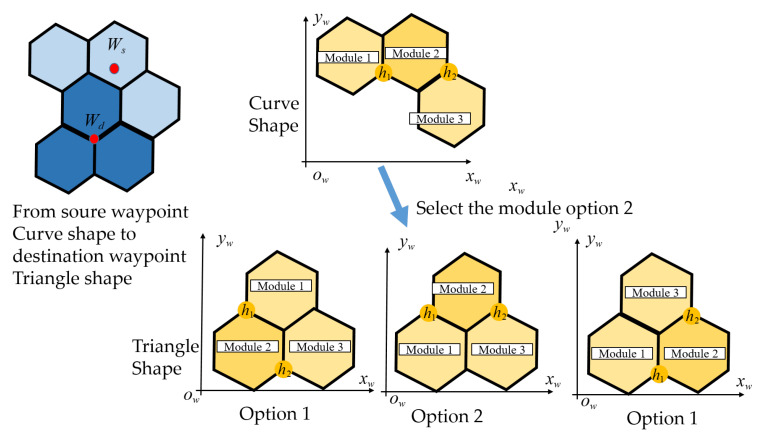
Selection of Module location of hTrihex robot when shape shifting from Curve to Triangle.

Algorithm 1 is used to find optimal modules of hTrihex for a given tileset in the workspace with row *r* and column *c*. In the case where the current robot morphology *m* is symmetric (Triangle or Bar), the module positions of the morphology *m*-1 with the least heading correction with morphology *m* is selected. As a result, the costweight to shift the robot from source to destination is reduced. Equation (1) determines the block locations for morphology*m* by finding the argmin of orientation offset among the options Φ. Figure 10 shows a case of assignment a square with a Triangle-shaped from the knowledge of the previous module location of the Bar shape. The algorithm chooses the first option, as depicted in Figure 10, because it yields a similar module location as the preceding Bar shape header.

(1) $$\hat{m}=\underset{p\in \Phi }{argmin}\left(\right|\varphi_{h}^{m}-\varphi_{h}^{m-1}\left|\right)$$

**Algorithm 1** Module assignment for hTrihex
**1 Function** LOCATION modules given workspace and tilseset:**2** get{r,c}**3***r*←1,*c*←1,*m*←1**4**   **let all***r*,*r*←1, execute**5**     **let all***c*,*c*←1, execute**6**          **assign w(r,c)** is center of mass of morphology *m*:**7**               **if** morphology *m* is asymmetrical:**8**                    *Assign*: morphology *m* modules as as Curve shape**9**               **else if** morphology *m* is symmetrical:**10**                    *Conduct:* moving from m−1 to *m***11**                    *Identify:* morphology with the least heading correction**12**                    *Assign*: morphology *m* modules as Bar and Triangle shape**13**              **end****14**         **end****15**    **end**


### 4.3. Optimal Planning for Navigation

**Figure 11:**
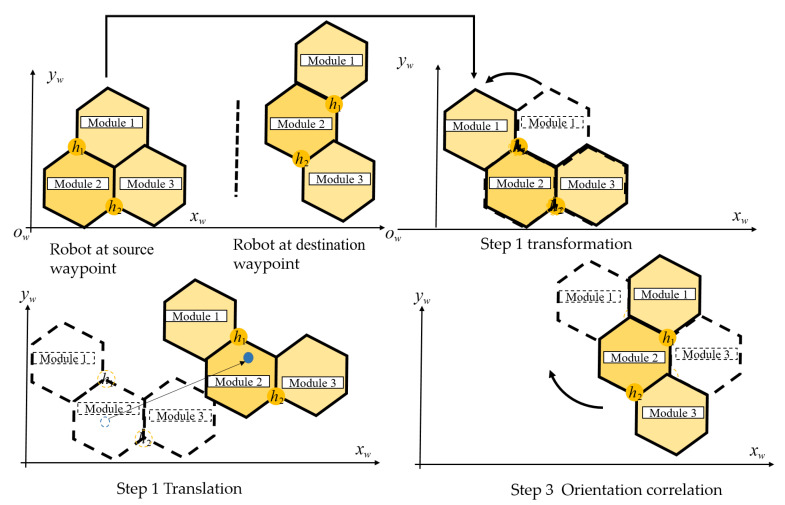
Three actions to move from source waypoint $W_{k}^{s}$ to a goal waypoint $W_{k}^{d}$.

The robot covers the workspace by clearing all pairs of predefined waypoints sequentially. Figure 11 shows the group of activities when the robot clears a pair of waypoint number *k* with a source waypoint $W_{k}^{s}$ to goal waypoint $W_{k}^{g}$. In particular, hTrihex performs three separate movement operations, including changing the shape to the desired shape of goal waypoint called transformation, linear movement from COM of source waypoint to COM of goal waypoint called translation, and pivot turn the whole robot to a particular goal waypoint heading inside the workspace called orientation correction. The turning angle $\theta_{i}$ of the robot module to change among three possible shapes is presented in Table 1. The turning of each module $l_{m}$ between the source and goal shapes is presented in Table 2. The heading adjustment is the difference between the robot direction at the goal waypoint heading $\varphi_{h}^{g}$ and the direction at source waypoint heading $\varphi_{h}^{s}$. As described in Figure 11, the robot direction stays static during shape-shifting, and then translates to goal waypoint and conducts the orientation correction.

The energy utilization while clearing the waypoint is derived by calculating the distance moved by either DC motors or servo motors, multiplied by the related mass of the robot module to complete each activity among transformation, translation, and orientation, as shown in Figure 11. Specifically, translation energy to carry each of the three robot blocks from source waypoint COM to goal waypoint COM proportions to the total summation of the 2D Euclidean displacements of all DC motors multiple by the corresponding mass of each module $m_{i}$, as in Equation (2). Transformation energy is found by multiplying the required rotation length $l_{m}$ and the mass of the corresponding module $m_{i}$ by $\phi_{2}$ of the module ID 2 and $\phi_{3}$ of the module ID 3 around the hinge joint of the hTrihex then adding the steering correction $\theta_{i}$ of three independently-controlled steering and driving Locomotion modules, as shown in Equation (3). Orientation energy is found by total summation of orientation offset to shift robot around the COR from source header $\varphi_{h}^{s}$ to goal header $\varphi_{h}^{g}$ multiplied by the mass and robot length $l_{m}$ of each of the three squares, as described in Equation (4). The total costweight of all three actions to clear the pair *k* includes the source $W_{k}^{s}\left(x,y\right)$ and the goal $W_{k}^{g}\left(x,y\right)$, as shown in Equation (5).

(2) $$E_{tranl}\left(W_{k}^{s},W_{k}^{g}\right)=\sum_{i=1}^{3}m_{i}\sqrt{{\left(x_{i}^{g}-x_{i}^{s}\right)}^{2}+{\left(y_{i}^{g}-y_{i}^{s}\right)}^{2}}$$

(3) $$E_{tranf}\left(W_{k}^{s},W_{k}^{g}\right)=\left(m_{2}\phi_{2}+m_{3}\phi_{3}+\sum_{i=1}^{3}m_{i}\theta_{i}\right)l_{m}$$

(4) $$E_{ori}\left(W_{k}^{s},W_{k}^{g}\right)=\sum_{i=1}^{3}m_{i}\left|\varphi_{h}^{g}-\varphi_{h}^{s}\right|l_{m}$$

(5) $$E\left(W_{k}^{s},W_{k}^{g}\right)=E_{tranl}\left(W_{k}^{s},W_{k}^{g}\right)+E_{tranf}\left(W_{k}^{s},W_{k}^{g}\right)+E_{ori}\left(W_{k}^{s},W_{k}^{g}\right)$$

(6) $$\hat{\rho }=\underset{k^{*}\in \Omega }{argmin}\sum E\left(W_{k*}^{s},W_{k*}^{g}\right)$$

**Figure 12:**
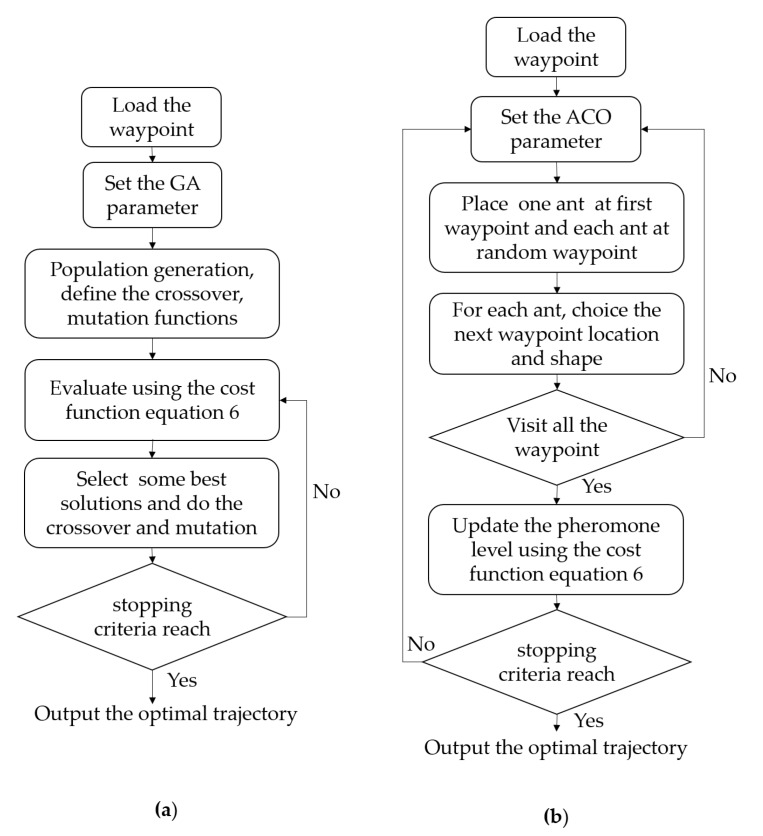
Block diagram for GA- and ACO-based TSP algorithms: (**a**) GA; and (**b**) ACO.

In this paper, we model the optimal solution ρ connecting all sets of waypoint pairs demonstrated according to Equation (6) as the NP-hard issue of the classic TSP optimization. To ease the complex nature of this issue, finding the Pareto-optima solution by probability approaches have been proposed in the literature. In this work, the ACO and GA-based evolutionary algorithms are utilized to address the TSP of sequencing navigation trajectory. The authors of [29,30] explained the motivations of GA and ACO and the mechanisms they execute to get the Pareto optimal solution for many nodes in the TSP. Both ACO and GA strategies implement the meta-heuristic loop to discover a better solution after each iteration, as described in Figure 12. Specifically, GA rehashes the determination and imitates steps to reject the unsatisfied qualities of chromosomes appearing after every iteration while preserving the valuable qualities in every population reproduction operations by mutation and crossover. Similarly, ACO utilizes the probabilistic way to deal with comprehension of the TSP by differing the choices of the ant agents at the waypoints and by continually updating the pheromones remaining in every integration. The meta-heuristic strategies do not ensure that the rout is the ideal global solution. The objective function for our CACP of hTrihex as in Equation (5) is the travel distance multiplied mass of all three modules to drive the robot from source waypoint to destination waypoint. Considering a workspace with *N* waypoints, the objective of the optimization in Equation (6) is to derive the shortest path to connect all the waypoints. The restrictions of this TSP optimization are that the robot has to start at initial source waypoint $W_{1}^{s}$ and, after reaching the final goal waypoint $W_{N-1}^{g}$, the robot no need to go back to the initial waypoint. These constraints will reduce the complexity of optimization. The evaluation process in GA and ACO will be launched to calculate the corresponding fitness values defined in Equation (5) for each agent of GA and ACO. The selection criteria will filter out individuals with weak performances. A new generation of the population will then be determined based on the encoded information in the remaining agents through operators such as mutation, crossover, and selection.

### 4.4. Execution Autonomous Area Coverage by hTrihex

**Figure 13:**
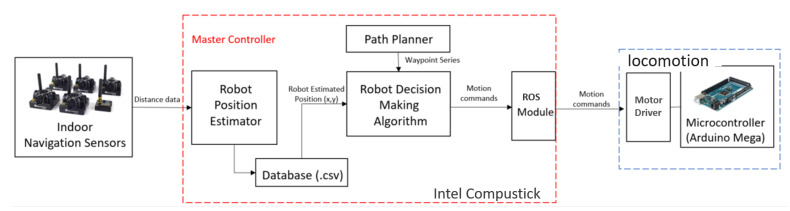
Flowchart of the robot motion planner for hTrihex.

After the CACP is found, the navigation process is started to cover the entire workspace with defined waypoint shapes and locations, as shown in Figure 13. The system built based on Robot Operation System [32] will arrange the reference waypoints *W*, including the location of the next goal and the desired shape in the order found from the CACP algorithm. During automatic movement, the robot will continuously refresh its current arrangement at reference points depending on the sensor input to determine which waypoints have been cleared and the remaining waypoints. After determining the position and shape of the next waypoint inside the workspace from the odomery’s position, the robot decides the actions to be conducted in the following order: transformation, linear movement, and heading correction to fit the robot in the required tile of the goal waypoint. If an inconsistency between the present hTrihex shape at pair *k* at the source waypoint $W_{k}^{s}$ and the following shape at the goal waypoint $W_{k}^{g}$ in trajectory is discovered, it will give an order to the robot microcontroller to execute a shape-shifting order by instructing the servo motors to rotate the predefined angle, as shown in Table 1. The robot’s present location $x_{h}^{w},y_{h}^{w}$ is always monitored to determine whether the difference value between robot location and desire location is lower than the threshold. If the condition is satisfied, the robot executes the navigation to the following defined waypoint. The same process is executed for the next waypoint until the waypoint queue is empty.

## 5. Experimental Results

The CACP frameworks were verified to yield the least costweight in simulated workspaces and the optimal energy consumption in the real environments.

### 5.1. Simulation Environment

**Figure 14:**
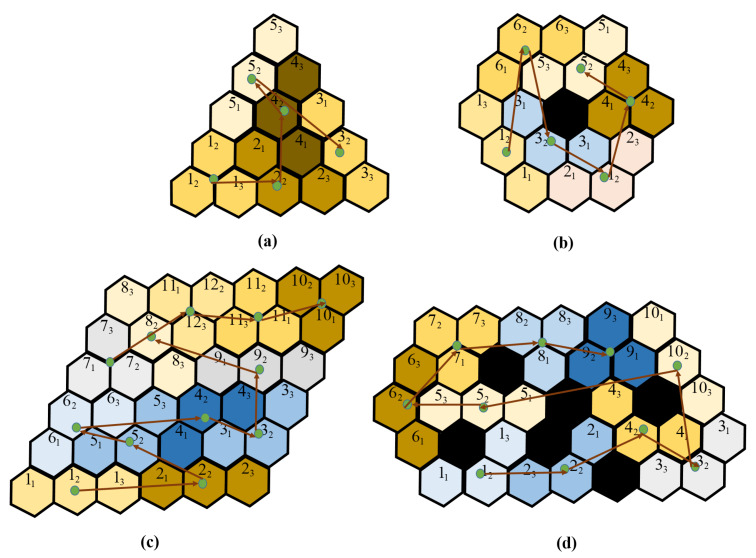
The ACO pareto-optimization trajectories: (**a**) triangle workspace; (**b**) round workspace; (**c**) rhombus workspace; and (**d**) workspace with obstacles.

Matlab Simulink was used to generate the simulated workspaces with different sizes, obstacle locations, and satisfying the tiling theory to ensure the complete coverage by the tileset consisting of the three robot shapes. The simulated workspaces were segmented into hexagon-based cells, and the cell has a shape similar to one module of the robot. The tilesets were generated for the tested workspaces by using backtracking algorithms [34]. The approaches of TSP, such as zigzag, spiral, greedy search, and evolutionary approaches ACO and GA, were used to generate the costweight for each workspace. Figure 14a–d are the workspaces with and without obstacle, respectively. The free space regions are colored differently based on the arrangement of robot modules inside the workspace derived by Algorithm 1. The obstacle regions located randomly are colored black, and the value was set as −1, which were ignored when generating the tilesets. Note that the workspace with obstacles was generated so that the tileset, including all three robot morphologies Triangle, Bar, and Curve, have to be used to cover it entirely and only one shape would fail to cover the space. The trajectories generated by the ACO algorithm for all testbed workspaces were denoted as red linking arrows. In the experiment, the best values of parameters were found through 100 experimental trials. The parameters for GA were: probability of mutation = 0.08 and chromosome counts = 200. The parameters for ACO were: probability of evaporation = 0.8 and ant agents = 200. The stopping criteria were set as the optimization costweight was not improved within 10 iterations or the optimization loops were over 1000 iterations. 

**Figure 15:**
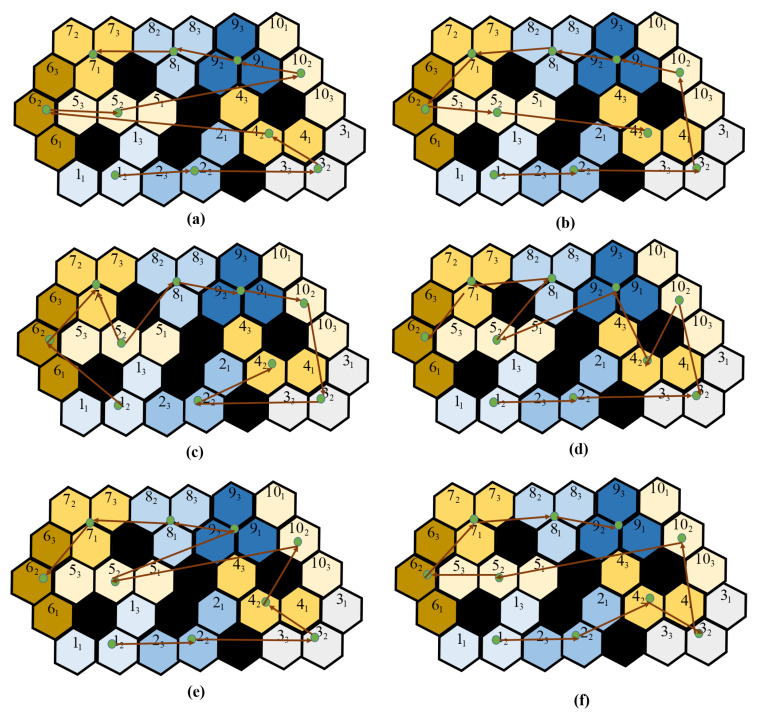
The trajectories comparisons of CACP approach: (**a**) zigzag scanning; (**b**) spiral scanning; (**c**) random search; (**d**) greedy search; (**e**) GA; and (**f**) ACO.

The tileset and the found trajectories for the workspace with obstacle is shown in Figure 15. The associated costweight, as well as the generated time of each method for this workspace, are presented in Table 3. All tested methods have similar Euclidean distances. Despite yielding the fastest generation time, the zigzag and spiral methods merely connect the waypoint in the simple row-wise and outer orders; their costweights are slightly higher than random search with 1000 iterations and greedy search methods and significantly higher than GA and ACO methods. With a significant amount of running time, the greedy search and random search do not guarantee the found paths being the best costweight. On the other hand, the evolutionary-based methods GA and ACO consumed less time for trajectory finding than the greedy search. As a result of evolutionary-based optimization, the costweights of the ACO are the least in comparison with the costweight of other methods.

When the trajectory found by GA and ACO have two waypoints with the same morphology, the one with less orientation correction is chosen, as shown in Figure 15e,f. For instance, from the waypoint 1 the proposed CACP by the evolutionary algorithms routes to waypoint 2 with less heading correction in rad instead of waypoint 6 with 5π/3 heading correction. Moreover, the evolutionary algorithms choose the route to the next tile without transformation, or the next tile with only one module needing to be rotates in rad a value of 4π/3 rather than two modules needing to be rotated the same value of 4π/3 such as from Triangle to Curve rather than Triangle to Bar. As a result of reducing the transformation and orientation steps when clearing all pairs of waypoints with the predefined workspace, the lowest costweight is archived by the proposed CACP with evolutionary-based methods.

**Figure 16:**
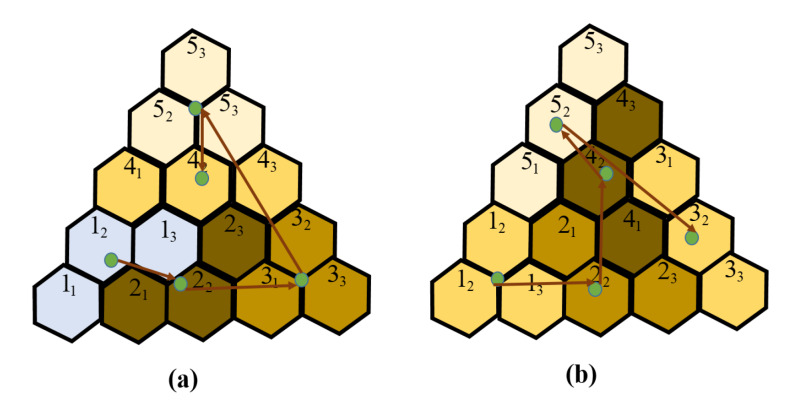
The optimal trajectories of CACP for two tileset in same workspace: (**a**) optimal sequence with costweight 21.19 Nm; and (**b**) optimal sequence with costweight 21.61 Nm.

Different tilesets associated with costweight are provided to tile the same workspace with the size are depicted in Figure 16. From the associated costweight, the navigation frameworks select the optimal tileset among the available tilesets to conduct the CACP of the given workspace entirely.

### 5.2. Real Environment Testbed

**Figure 17:**
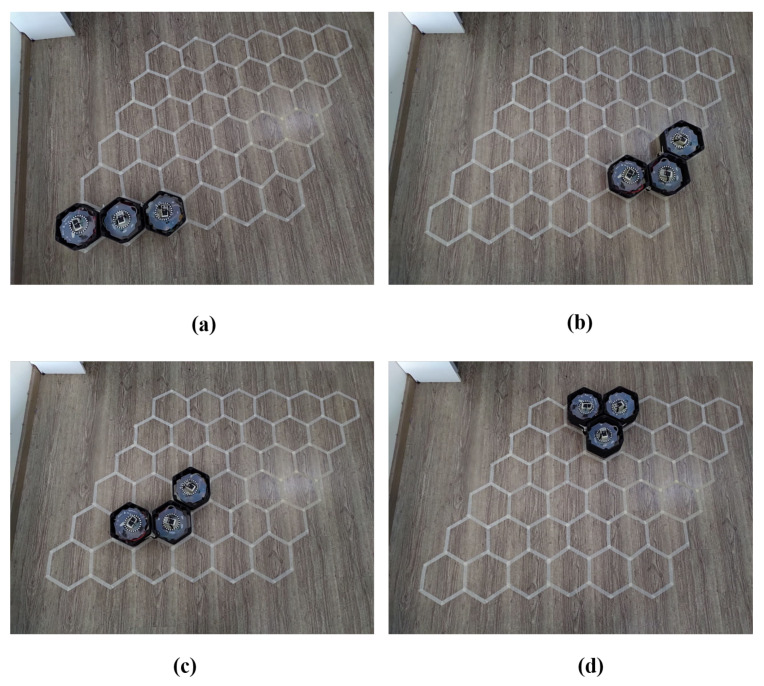
Real tested workspace with 12 tileset as in the simulated workspace in Figure 14c: (**a**) robot at waypoint 1; (**b**) robot at waypoint 5; (**c**) robot at waypoint 7; and (**d**) robot at waypoint 9.

**Figure 18:**
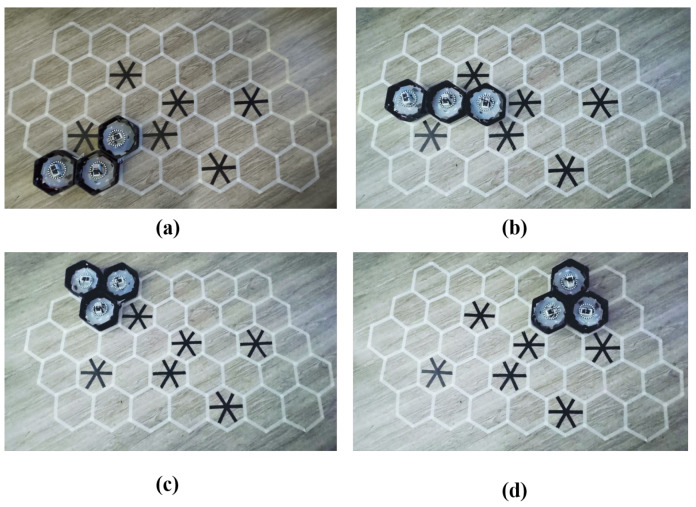
Real tested workspace with obstacles as crossed cells as in the simulated workspace in Figure 14d: (**a**) robot at waypoint 1; (**b**) robot at waypoint 5; (**c**) robot at waypoint 7; and (**d**) robot at waypoint 9.

The energy spent to complete the autonomous navigation following the trajectories found in the simulated workspace was verified in a real test bed. Snapshots of navigation sequences for the workspace in Figure 14c,d are shown Figure 17 and Figure 18, respectively. The robot was set to automation mode and clear edone by one the waypoints at the robot COM stored in the database, which includes the 2D locations and desired shapes. The actions transformation, translation, orientation correction were done orderly. The system operated under the monitor of the ROS system. The moving command generated by the PID controller was sent to motor drivers to issue appropriate velocity for the DC motors and desired rotating angles of the servo motors at the robot hinges for shape change, the steering servo motor to change the directions steering units, and the DC motors for linear moving. The real-time robot localization [35] enhanced by extended Kalman filter, EKF multiple sensors fusion, the industrial UWB system, wheel encoder, and IMU made sure the robot understood the position even in the cases any sensor malfunctioned. The power consumption of the hTrihex was calculated by reading the current sensors connected with the main robot battery power supply (14.4 V, 1000 mAh). The frequency of the operation current was sampled at 10 kHz and 7 V. The DC motor was set with a maximum speed of 50 rpm.

The energy speeding, travel time of the all tested method are presented in Table 4. From the given values, if the robot takes the trajectory of the method which yields the smaller costweight value, the final energy consumption will be smaller. Specifically, the zigzag consumes the highest power, followed by spiral. The best CACP method in terms of saving power consumption and time spent to fulfill the trajectory is ACO. ACO yields about 30% less than the greedy search as the third method. The results prove that the proposed CACP is a feasible method for area coverage by the tiling hTrihex robot.

The energy for single actions among transformation, translation, and orientation correction to finish the tested trajectories are depicted in Table 4. Translation consumes the most energy since all three DC motors have to carry the whole robot mass, and all steering servos motors are steering to correct heading. Transformation takes the second most energy, with the energy consumption of orientation in third place. We can observe in the workspace at tile 5 in Figure 15b for the narrow space, the robot needs to change to the Bar shape to navigate through the narrow space created by obstacles.

Complex mechanical architecture of hTrihex raises the challenges to control issues and analytical-based energy consumption estimation. In this paper, we assume that the summation of travel distances multiplied by the robot block mass of all three modules to navigate the robot from source to goal waypoints is proportional to the consumed energy based on three actions: transformation, translations, and orientation correction. As the kinematic control is implemented in the current platform, for which motion is slow and the mass is small, we ignored the dynamic part of the platform. Although the implementation aspect is simple due to trigonometric equations, the current approach results in a simple and practical solution to approximate the energy consumption in which voltage is regulated and the overall current drawn varies insignificantly during transformation, translations, and orientation correction. Thus, energy consumption is directly proportional to the distance traveled, assuming that slippage is also negligible. Now, the platform can work only on simple workspace. We are developing hTrihex to be able to work autonomously in a wider testbed environment with complex obstacles. Once the stable platform has been constructed, evolutionary algorithms with different parameters setting will be evaluated to identify the best optimization technique that yields the ideal results.

## 6. Conclusions

The proposed reconfigurable hTrihex with three available honeycomb-based morphologies provides a feasible solution to tile the multiple predefined workspaces. The evolutionary-based complete path planning for the hTrihex was validated in both simulation and real environments and more efficient in terms of energy-saving and time consumption than the conventional CACP methods. The proposed CACP can be applied to other tiling robot platforms. The research is the first step to make the proposed tilling-based platform a commercial cleaning product. The research opens several potential studies that need to be conducted, such as control and autonomous strategies. Future research work can be extended to follows: (1) energy estimation model in the dynamic and cluster workspaces; (2) studying locomotion with trajectory tracking and following while performing the tiling motion on the generated tileset; (3) applying tiling theory under the cellular decomposition technique where we apply different tiling theory for each decomposed cell in a complex environment; (4) optimal tileset generation by learning-based approaches; (5) studying long-term autonomy with the tiling motion on a physical tilling robot platform; and (6) further analyses on the energy consumption of the electrical parts, robot motion, and frictions. 

## Figures and Tables

**Table 1:** The hTrihex module’s rotating $\phi_{i}$ degree to shift the robot shape from source waypoint $W_{k}^{s}$ to a goal waypoint $W_{k}^{g}$.

	$W_{k}^{g}$	Triangle Shape	Bar Shape	Curve Shape
$W_{k}^{s}$		$M_{1}$ $M_{2}$ $M_{3}$	$M_{1}$ $M_{2}$ $M_{3}$	$M_{1}$ $M_{2}$ $M_{3}$
Triangle Shape		0 0 0	4π/3 0 4π/3	0 0 4π/3
Bar Shape		−4π/3 0 −4π/3	0 0 0	0 0 4π/3
Curve shape		0 0 −4π/3	0 0 −4π/3	0 0 0

**Table 2:** The rotating length of each module of hTrihex to shift the robot shape from source waypoint $W_{k}^{s}$ to a goal waypoint $W_{k}^{g}$.

	$W_{k}^{g}$	Triangle Shape	Bar Shape	Curve Shape
$W_{k}^{s}$		$M_{1}$ $M_{2}$ $M_{3}$	$M_{1}$ $M_{2}$ $M_{3}$	$B_{1}$ $B_{2}$ $B_{3}$
Triangle Shape		0 0 0	$l_{m}$ 0 $l_{m}$	0 0 $l_{m}$
Bar Shape		$l_{m}$ 0 $l_{m}$	0 0 0	0 0 $l_{m}$
Curve shape		0 0 $l_{m}$	0 0 $l_{m}$	0 0 0

**Table 3:** Costweight and generation time of CACP approaches.

Approach	2D	Total Cost	Running
	Distance (m)	Weight (Nm)	Time (Second)
Zigzag	10.93	93.33	0.01
Spiral	10.99	94.37	0.05
Random search	10.33	84.1	31.34
Greedy search	10.42	81.36	35.25
Propped method GA	10.12	61.99	1.25
Proposed method ACO	10.23	61.35	1.22

**Table 4:** Averaged energy and time evaluation for realtime testbeds. The measured electrical energy in (Ws) unit has been converted to (J) unit to describe the mechanical energy.

Method	Costweight	Summation	Translation	Transformation	Orientation	Travel
-	(Nm)	Energy (J)	Energy (J)	Energy (J)	Energy (J)	Time (Second)
Zigzag	103.19	20.41	9.19	6.52	4.70	251
Spiral	104.45	19.82	9.32	6.71	3.79	227
Random search	94.28	18.44	8.69	5.86	3.89	202
Greedy search	91.52	17.56	8.52	5.13	3.91	204
GA	69.83	12.59	7.21	3.21	2.17	198
ACO	68.97	12.33	7.46	2.96	1.91	193
